# CD34 Over-Expression is Associated With Gliomas’ Higher WHO Grade

**DOI:** 10.1097/MD.0000000000002830

**Published:** 2016-02-18

**Authors:** Xiangyi Kong, Jian Guan, Wenbin Ma, Yongning Li, Bing Xing, Yi Yang, Yu Wang, Jun Gao, Junji Wei, Yong Yao, Zhiqin Xu, Wanchen Dou, Wei Lian, Changbao Su, Zuyuan Ren, Renzhi Wang

**Affiliations:** From the Department of Neurosurgery, Peking Union Medical College Hospital, Chinese Academy of Medical Sciences, Beijing, P. R. China.

## Abstract

CD34 is a transmembrane phosphoglycoprotein that was first identified on hematopoietic stem and progenitor cells. CD34 is known as an optimum marker for microvascular density studies and it is positively stained in pathological and physiologic vessels. The use of CD34 for the prognosis, diagnosis, and treatment of neoplasms has been increasingly discussed. The implications and utilities of CD34 in WHO grades of gliomas and its prognosis have been reported rarely. Also, the WHO grades and prognosis researches remains unclear and controversial. A meta-analysis is the best choice for drawing a convincing conclusion.

Several databases were searched. We carefully assess the relevant articles and standard mean differences (SMDs) with 95% confidence intervals (95% CIs) were estimated in terms of the relationship between CD34 expression levels with gliomas’ WHO grades, patients’ ages and gender. We used the Galbraith figure, the I^2^ test, and Cochran Q test to evaluate the heterogeneity of the included studies. A sensitivity analysis was conducted to assess the pooled results’ stability. A Contour-enhanced funnel plot evaluation was made to assess potential publication bias. Ethics review and approval was not necessary because the meta-analysis did not involve any direct human trials or animal experiments.

There were 12 eligible studies, including 684 patients who were considered in the present meta-analysis. All of them were conducted in China. CD34 overexpression in glioma tissues was associated closely, according to the pooled SMD, with higher WHO grade (III + IV) (SMD -1.503, 95% CI -1.685 to -1.321; *P* = 0.000). There were no significant associations between CD34 and age (SMD -0.223, 95% CI -0.602 to 0.156; *P* = 0.248) and CD34 and gender (SMD -0.059, 95% CI -0.439, 0.321; *P* = 0.761). No publication bias was detected according to Contour-enhanced funnel plot.

Our results suggested that CD34 overexpression is associated with higher WHO grades of gliomas. CD34 may serve as a potential diagnostic and prognostic marker, or it could be a useful therapy target.

## INTRODUCTION

Glioma is a common central nervous system tumor. Surgical resection is the usual treatment and chemotherapy and radiotherapy are important adjuvant therapies. Gliomas can be classified into 4 pathological grades (I–IV) on the basis of tumor pathological classification by WHO.^1^ Grade I gliomas are generally considered as relatively benign and potentially curable. Grades II∼IV gliomas, or more specifically, Grades III and IV, are usually aggressive malignancies with a tendency of infiltration and invasiveness.^2^ With the WHO grade system, surgeons can treat glioma patients as early as possible. It is therefore necessary to identify a helpful and valuable biomarker to grade tumors and predict their prognosis.

CD34 was originally regarded as a marker of hematopoietic stem cells (HSCs) and hematopoietic progenitor cells.^3^ However, CD34 is now also established as a marker of many other nonhematopoietic cell types, including vascular endothelial progenitors and embryonic fibroblasts.^4,5^ The role of CD34 in the diagnosis, prognosis, and treatments of tumors has been increasingly discussed, such as liver cancer, breast cancer, ovarian cancer, pancreatic cancer, and urothelial cancer.^6–9^ CD34 expression has been demonstrated to play a crucial role in the regulation of glioma angiogenesis by promoting a new blood vessel network and fueling further glioma growth. This occurs by the increased supply of oxygen and other necessary metabolites, which leads to brain invasion and a worsened prognosis.^10–12^

The exact role of CD34 in grading and determining a prognosis for gliomas, however, is still unclear and under debate. Rahmah et al^11^ found that high-grade gliomas showed higher expression of CD34, which suggested that high-grade glioma may have higher densities of vessel, whereas Netto et al^13^ found high expression levels of CD34 in the endothelial cells in their oligodendroglioma study. They did not find any significant correlation to the grades of oligodendroglioma.^13^ We carried out a meta-analysis to make clear the practical significance of CD34 and resolve the between-study heterogeneity. This is possible because a meta-analysis is able to avoid the potential bias of sources such as with the selected populations and study methodology. In our study, we extracted data from published articles and systematically assessed CD34 regarding gliomas’ WHO grades and prognosis.

## METHODS

### Search Strategy

We used PubMed, Google Scholar, Ovid, the Cochrane Library, EMBASE, Wanfang and Cnki databases with a cutoff date of April 2015. We did not restrict sources and languages. We used the search terms “CD34,” “gliomas [MeSH],” “expression,” “grading,” “grade,” etc. We scanned all the retrieved papers to identify other potential studies.

### Study Selection

The studies were chosen independently by 2 reviewers. If there was any conflict between the 2, the third person would be used to solve the problems. The including criteria of the research were listed as follows: the principle outcomes of the study focused on pathological grade, gender, age, OS, etc.; pathologists were responsible for giving the diagnoses of gliomas with their grades; upon immunohistochemistry (IHC), the microvascular density of a positive CE34 stain (CD34-microvessel density [MVD]) and SD could be directly extracted from the literatures or calculated according to the graphs or tables provided by the study report; a CD34 expression model was identified by IHC methods or other dependable molecular pathological methods; and in the case of duplicate studies, only the most comprehensive and the most recent one could be involved in the analysis. Contacting with the authors would also work the same.

### Data Extraction

A purpose-designed form was used for data collection by 2 reviewers independently. First author name, country, publication year, histological results, study means, WHO grades, the number of the patients, mean age were recorded. A third reviewer resolved any disputes between the 2 initial reviewers.

### Quality Assessment

Two individual reviewers ran their data evaluations through ELCWP, in which they assessed the quality scales of all sources.^14^ The highest score is 10 for 1 factor so that the highest total score for 1 source is 40. A third intermediate person would solve any disagreement between 2 initial reviewers. The final scores were given by dividing the actual score by 40. Therefore, the scores, also expressed as percentage forms of the maximum achievable scores, determined the quality of the sources.

### Data Synthesis and Analysis

Differences are indicated in the form of SMDs (95% CI). STATA 12.0 (StataCorp LP, College Station, TX) was used for all statistical analyses. Cochran Q test, the I^2^ test (variation in SMD attributable to heterogeneity), and a Galbraith figure were used to judge heterogeneity among the included studies.^15,16^ If all the circles in the Galbraith figure are distributed in an area enclosed by the upper line and the lower line, then there is evidence for homogeneity. Also, if *P* < 0.10 for the Q statistic, then heterogeneity was considered to be statistically.^17^ I^2^ values of 25%, 50%, and 75% were considered as evidences of low, moderate, and high heterogeneity, respectively.^15^ A fixed-effects model shall be used when there were no evidences of statistical heterogeneities among the literatures. Random-effects models (DerSimonian and Laird) were used otherwise.^17^

A sensitivity analysis was carried out by virtue of a one-at-a-time way to assess the pooled results’ stability. This method omits 1 study at a time and then repeat the meta-analysis. If omitting 1 study obviously changes results, it suggests that the pooled result was sensitive to this study. Visual inspection of the Contour-enhanced meta-analysis funnel plots was used to assess potential publication bias. Asymmetry in the plots, which may be due to studies missing on the left-hand side of the plot that represents low statistical significance, suggested publication bias. If studies were missing in the high statistical significance areas (on the right-hand side of the plot), the funnel asymmetry was not considered to be due to publication bias.^18^ Meta-regression analyses were not made because the included studies were not large enough.

There were 2 sides for all *P* values, and it was seen as an extremely large number when *P* < 0.05. STATA 12.0 (StataCorp LP) was used for all statistical analyses.

## RESULTS

### Characteristics of Studies and Search Results

**FIGURE 1:**
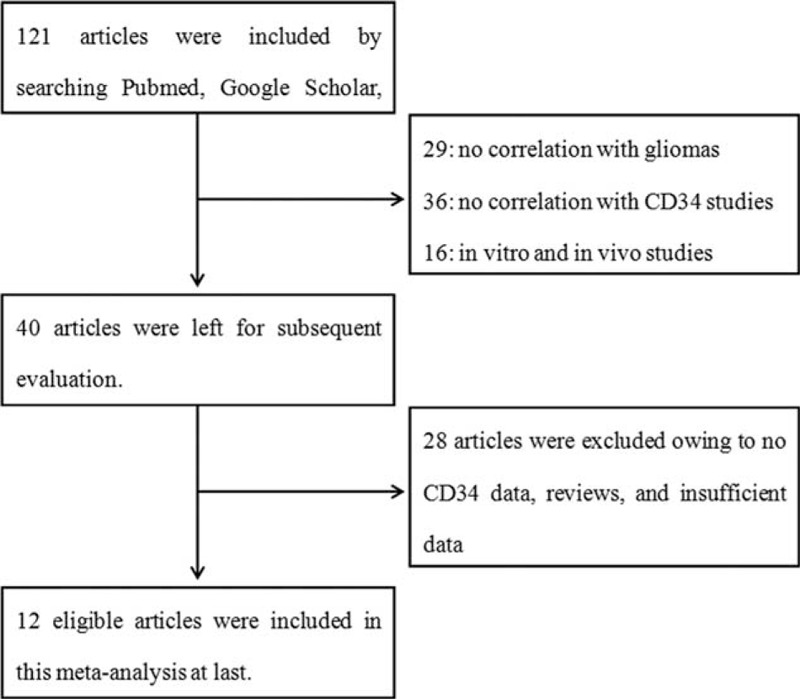
Selection of articles based on the literature search.

Figure 1 shows the article search process. Initially, the literature search from multiple databases produced 121 articles. Of these, 81 were excluded on the basis of the title and abstract: 29 had no relevance with gliomas, 36 had no relevance for CD34 studies, and 16 were in vivo or in vitro studies. The remaining 40 literatures were assessed again and 28 articles were excluded because there were no CD34 data, they were reviewers, or had insufficient data. All of the included articles were from China. Finally, 12 articles were determined to have met inclusion criteria and were included in this study. Table 1 summarizes the general characteristics of these studies. There were 684 patients in the studies with a mean age of 42.1 years and there were 346 cases of low-grade gliomas (I + II). All 12 studies included Chinese patients. CD34 staining was counted as MVD, and manual counting was used to quantify the positive foci for slides counterstained with hematoxylin. CD34-MVD varied from 3.84 to 64.6. All 12 studies offered WHO grade data, 2 studies analyzed the correlation between CD34 and age and gender, 1 also studied the factor of tumor size, and the other studied the correlation between CD34 and tumor location, whether there was relapse or not, and it was not dependent on survival time.

**TABLE 1:**
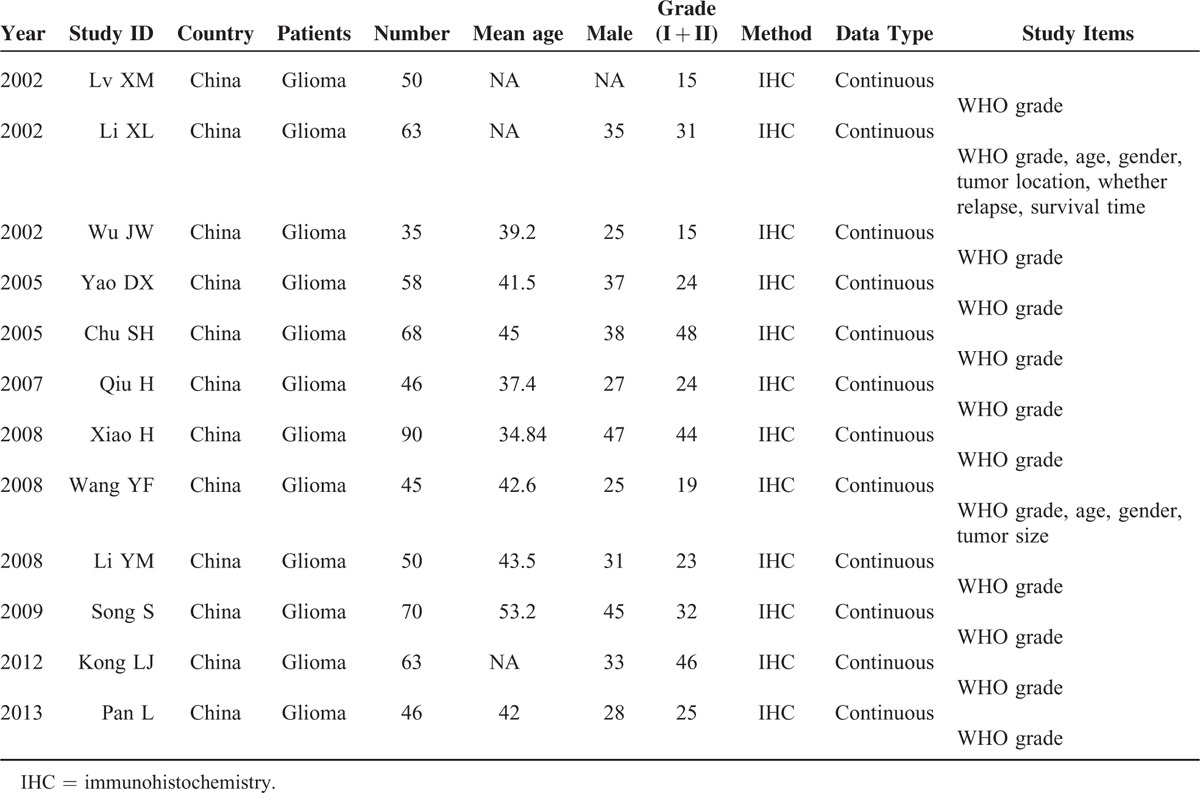
The Characteristics of the 12 Included Articles

### Study Quality

The quality of the study was assessed on the basis of the putative ELCWP. Table 2 summarizes the detailed scores. The mean global score for these studies was 79.75%. For all these studies, the mean score was 8.23 for the results analysis, compared with the design score (7.77), method score (8.04), and generalizability score (7.87). The average global score of the 10 articles containing only WHO grade studies was 79.7% and the mean score of the remaining 2 articles was 80.0%. There was no statistical difference between the scores on the basis of Student *t* test, which indicated that baseline characteristics among different groups had homogeneity.

**TABLE 2:**
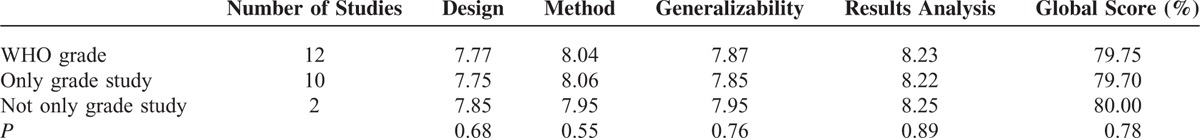
Clinical and Methodological Characteristics of 12 Included Studies

### A Meta-analysis on CD34, WHO Grades, and Prognosis

**FIGURE 2:**
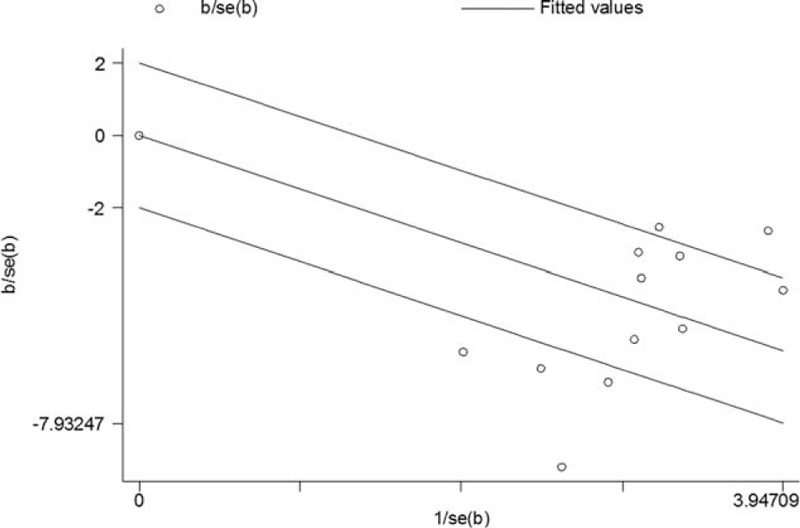
The Galbraith figure including the studies that focused on the correlation between CD34 expression levels and WHO grades. Circles distributed within the region bounded by the upper and lower lines were taken as evidence of homogeneity. If the circles were farther away from that region, it indicated heterogeneity.

**FIGURE 3:**
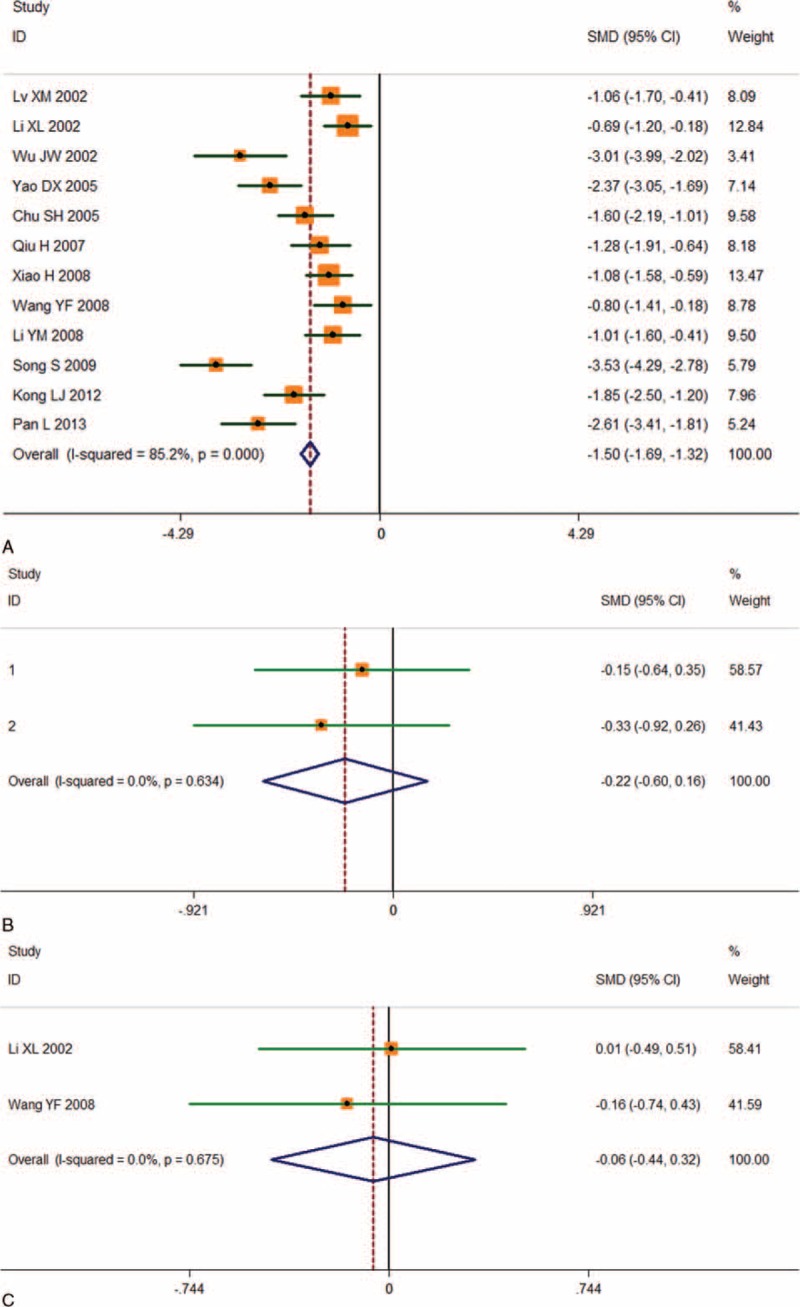
Individual and pooled SMD results with 95%CI for CD34 and WHO grades, age, and gender. The random-effects model analysis showed an association between CD34 and WHO grades (n = 12, SMD -1.503, 95% CI: -1.685 to-1.321; *P* = 0.000) (A). There was no significant association between CD34 and age (SMD -0.223, 95% CI: -0.602 to 0.156; *P* = 0.248) (B) and gender (SMD -0.059, 95% CI: -0.439 to 0.321; *P* = 0.761) (C).

The WHO grades were classified into high grades (III + IV) and low grades (I + II) for the crosstable. Table 1 summarizes the clinical grades for all 12 studies, which included continuous data. The Galbraith figure (Figure 2) shows that half of the points fell outside the appointed region. This is evidence for heterogeneity among the studies (Q = 74.3, d.f. = 11, I^2^ = 85.2%). As shown in Figure 3A, using a random effects model, a significant association between CD34 overexpression and higher WHO grades was revealed by the SMDs (SMD -1.503, 95% CI -1.685 to -1.321; *P* = 0.000), which indicated that there was higher CD34 expression in the postoperative glioma tissues than normal cerebral tissues and this could be used to predict gliomas of high grades. High CD34 expression levels are recommended as a valuble marker for diagnoses. The heterogeneity was not significant for age (Q = 0.23, d.f. = 1, I^2^ = 0.0%) and gender (Q = 0.18, d.f. = 1, I2 = 0.0%; figures not shown); therefore, a fixed effect model was selected. No significant associations between CD34 and age (Figure 3B, SMD -0.223, 95% CI -0.602 to 0.156; *P* = 0.248) and CD34 and gender (Figure 3C, SMD -0.059, 95% CI -0.439 to 0.321; *P* = 0.761) were detected.

There were no articles that summarized high CD34 expression levels and overall survival.

### Publication Bias and Sensitivity Analysis

**FIGURE 4:**
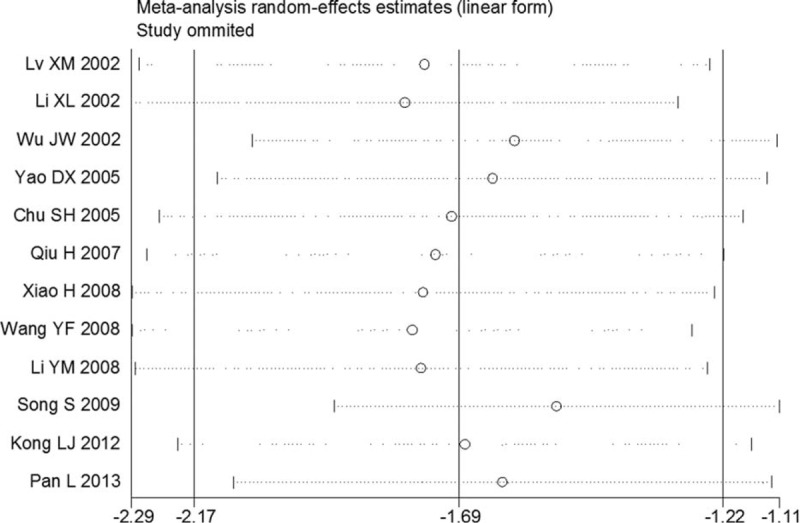
Sensitivity analysis for the 12 included studies. Each article was omitted in turn and the result was computed. A meta-analysis random-effects estimate (exponential form) was obtained. The ends of the dotted lines represent the 95%CI.

**FIGURE 5:**
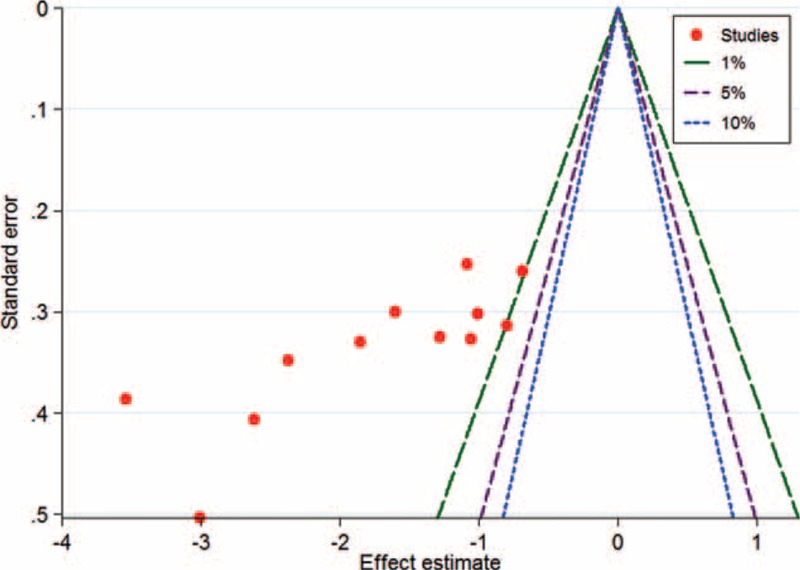
A contour-enhanced meta-analysis funnel plot was created to show potential publication bias. If studies appear to be missing on the right-hand side of the plot, then publication bias is not the cause of funnel asymmetry.

The effects of each single study on pooled SMDs were evaluated using the sensitivity analysis, in which each study was taken out of consideration during the assessment (Figure 4). The outcome showed that the pooled SMDs for CD34 and WHO grade were not obviously influenced by any single study, suggesting a robust result. Publication bias analysis of these 12 reports was conducted with a Contour-enhanced meta-analysis funnel plot, showing that the studies had missing areas for high statistical significance (in the right-hand side of the plot). This suggested that the funnel asymmetry was less likely to be led to by publication bias (Figure 5).

## DISCUSSION

Recent movements toward antibody-based therapeutic strategies in cancers have resulted in the characterization of several potential antigens. CD34 is one such antigen gaining widespread popularity. CD34 is a member of the single-pass transmembrane sialomucin protein family. This family of proteins expresses on early hematopoietic and vascular-associated tissues.^3^ Little is known about the exact functions of this family of proteins. CD34 is found in the alveolar soft part of sarcomas, preB-acute lymphoblastic leukemia (ALL) (positive in 75%), acute myeloblastic leukemia (AML) (40%), AML-M7 (most), gastrointestinal stromal tumors, dermatofibrosarcoma protuberans, giant cell fibroblastomas, granulocytic carcomas, Kaposi sarcomas, liposarcomas, malignant fibrous histiocytomas, meningiomas, malignant peripheral nerve sheath tumors, mentingeal hemangiopericytomas, schwannomas, neurofibromas, and papillary thyroid carcinomas.^5^ In recent years, studies have demonstrated that CD34-positive vessels play pivotal roles in the development of gliomas and have an influence on the malignant severity.^10,11,13^ There have been a few reports that have comprehensively described the significance of CD34 in glioma patients and the specific roles of CD34 in the grading and prognosis of gliomas remain unclear. Therefore, it is unclear whether CD34 can be used as a marker of WHO grades in gliomas. In our study, we used PubMed, Google Scholar, Embase, Cnki, and Wanfang to systematically evaluate clinical effects of CD34.

CD34 expression was reported in 12 glioma studies and the association with WHO grades in 684 patients was investigated. We assessed the literature quality according to ELCWP and found no significant differences among the studies. A random-effects model was used when there was significant heterogeneity to determine the pooled SMD. This provided a more conservative standard error and a larger confidence interval. CD34 expression in glioma tissues was shown to be closely associated with high WHO grades (III + IV; SMD -1.503, 95% CI: -1.685 to -1.321; *P* = 0.000). There were no significant correlations between CD34 and age (*P* = 0.248) or gender (*P* = 0.761).

To assess the heterogeneity among the included studies, Cochran Q-test, a Galbraith figure, and the I^2^ test (variation in SMD attributable to heterogeneity) were used. Heterogeneity was considered statistically significant when *P* < 0.10 and/or I^2^ > 50%. There was heterogeneity in the studies regarding WHO grades in this meta-analysis. We therefore used a random-effects model. Several limitations in this study should be mentioned. CD34 expression in the literatures was investigated only in Chinese population and was tested using different criteria and methods. In particular, IHC was mostly dependent on methodological factors, which included primary and secondary antibody titers. It was difficult to carry out subgroup analyses using different antibodies to explore the potential bias of the methods for the pooled results. Also, most of the studies did not report complete data, although this may not have affected the bias.

A principle concern for meta-analysis is publication bias.^19^ Most studies report positive outcomes and negative results are usually not reported. In our study, we adopted the method of Contour-enhanced meta-analysis funnel plot to evaluate potential publication bias. Contours of statistical significance on a contour-enhanced funnel plot are overlaid. When contours of statistical significance are added, it facilitates evaluations of statistical significance and whether the areas in which studies are missing actually correspond to areas of low statistical significance. In general, when studies are missing in the area of low statistical significance, then the asymmetry may be due to publication bias. On the contrary, if the areas wherein studies are perceived to be missing are of high statistical significance, the publication bias is less likely to be the cause of funnel asymmetry.^18^ Therefore, in the present meta-analysis, the funnel plot indicated no publication bias. However, of note, the languages of the included articles were either English or Chinese, which means that the studies in other languages that met the including criteria may have caused publication bias.

In conclusion, we identified CD34 as being extremely associated with high WHO grades in our meta-analysis. This means that CD34 could be a potentially prognostic factor of gliomas. Of most importance, a pathological CD34 gene or protein could provide new insight into accurate predictions and early regimens for patients undergoing surgical resection.
